# Flexural and Pseudo-Ductile Performance of Unidirectional and Bidirectional Carbon Fabric-Reinforced Mortar

**DOI:** 10.3390/ma18050949

**Published:** 2025-02-21

**Authors:** Samy Yousef, Regina Kalpokaitė-Dičkuvienė, Sharath P. Subadra, Stasė Irena Lukošiūtė

**Affiliations:** 1 Department of Production Engineering, Faculty of Mechanical Engineering and Design, Kaunas University of Technology, LT-51424 Kaunas, Lithuania; 2 Laboratory of Materials Research and Testing, Lithuanian Energy Institute (LEI), Breslaujos 3, LT-44403 Kaunas, Lithuania

**Keywords:** carbon fabric, cement mortar, pseudo-ductile behaviour, ductility index, flexural response

## Abstract

This research aims to study the effect of introducing unidirectional (CFu) and bidirectional (CFb) carbon fabric into cement mortar (CM) on its flexural and pseudo-ductile performances. The experiments were performed on fabric/CM samples with a varying fabric distribution (single, double, and triple layers). The cohesion of fabrics in CM matrices and morphology of the damaged surfaces were examined using an optical microscope, while the flexural response was measured using a universal testing machine. The pseudo-ductile property, in the form of the ductility index (DI), was numerically modelled for CM matrices based on the measured flexural curves using different energy criteria models. Microstructure analysis showed a strong fabric cohesion in the matrices along with the production of more hydration products, which led to a transformation in the linear load–deformation relationship of mortar into the ideal shape of ductile material in the case of CFb/CM. In the case of the CFu/CM samples, two main drop points appeared with a long distance between them. In addition, the flexural load was significantly increased by introducing three layers of each type of fabric to CM, with an improvement of 75% (CFu/CM) and 68% (CFb/CM) compared to neat mortar. Similarly, the deformation till break was improved by 452% (CFu/CM) and 367% (CFb/CM). The DI analysis confirmed these results: the DI performance was improved by up to 140% by embedding. Based on these results, carbon fabric has high potential to enhance the strength and ductility of cementitious matrix.

## 1. Introduction

Recently, fabric-reinforced cementitious matrix (FRCM) or textile-reinforced mortar (TRM) has received great attention from researchers and manufacturers. Also, it began to be dealt with as a new generation of construction and building materials free of rebar and its disadvantages [1,2]. Steel bars lose many of their characteristics when operating in aqueous and chemical environments due to the corrosion phenomenon, followed by, in many cases, collapse, destruction, and inevitable losses in coastal structures [3,4]. In addition, steel bars have modest behaviour in cases of dynamic loads resulting from extreme proceedings [5]. Typically, in this type of FRCM composite, one or several layers of fabrics are included in order to provide the matrix with more strength, ductility, toughness, and energy absorption [6,7,8]. The use of fabric in the matrix creates multiple cracking criteria that allow for failure delay and prediction [9]. In addition, the high thermal, mechanical, and chemical resistance of fabrics allows them to be combined with CM for use in many specific and critical applications and environments, including deserts, sea water, warm temperatures, coastal areas, and areas subject to dynamic loads like explosions and earthquakes, in order to maintain the structural integrity of buildings [10,11,12,13]. Also, fabrics can easily be distributed uniformly in arrays, in contrast to chopped fibres and short fibres (as a single filler or hybridization) [14,15], which, in some cases, provides random dispersion in the matrix, resulting in random properties of matrices. This problem always occurs when fabrics are used on an industrial scale [16]. In addition, this failure still happens due to brittle fractures with similar features [17]. All these features are a source of concern for designers and manufacturers because the facture occurs suddenly, without any warning, and in a catastrophic manner. Thus, a higher safety factor must be applied in order for the product to remain safe, and this means taking on an additional cost [18].

In the literature, several studies have investigated the effect of types of yarns and of fabric design and structure on the bonding of fabric in the matrix and the impact of this effect on other properties, including flexural behaviour [19,20,21]. Fabric can also be composed of a huge amount of yarn distributed uniformly or heterogeneously in one direction (unidirectional fabric) or in two directions (bidirectional fabric) with a different weaving structure [22,23]. These yarns are made from different materials and fibres: glass and carbon fibre are the most common types [24,25,26]. However, carbon fibre demonstrates higher mechanical performance compared to glass fibre [27]; therefore, carbon fibre was used in this work. The results showed that increasing fabric density led to an increase in the bending load at the failure zone, thereby increasing the material’s capability to inhibit crack propagation. In addition, the composition of fabric has a significant effect on the properties of cementitious matrix, including increased first crack resistance and energy absorption of the matrix under the applied load. Also, the properties of cementitious composites, including compressive and flexural strengths, were modelled and predicted using an artificial neural network (ANN) and a holistic model [28]. Machine learning was also applied to predict the effects of introducing different types of fibres into cementitious composites [29].

**Figure 1:**
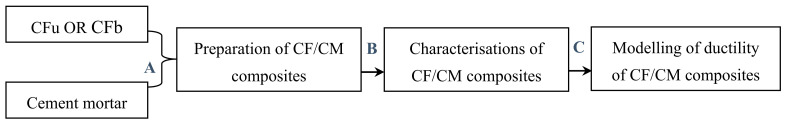
A workflow diagram of the current work.

On the contrary, recent studies showed that the reinforcement of cement with fabric can help to create pseudo-ductile phenomenon in the matrix, especially in the elastic region, thereby treating it as a ductile material [30]. This phenomenon can also be controlled by the distribution of fabrics in the matrix, by their structure, and by fabric mesh [31]. Unfortunately, there are not enough data in the literature about this phenomenon or about the influence of fabric on pseudo-ductile performance. Also, research on the woven carbon fabric type remains inadequate despite its high mechanical strength. In this context, this study was developed to investigate the flexural performance and pseudo-ductile behaviour, in terms of ductility index (DI), of carbon fabric-reinforced mortar. The matrices were prepared using two types of carbon fabric (unidirectional and biaxial) with different distributions in the matrix and different embedded fabric layers (single, double, and triple layers). Based on the measured flexural parameters, DI was calculated using two different models based on energy criteria [32], and a flowchart of the current work is displayed in Figure 1.

## 2. Experimental

### 2.1. Materials and Chemicals

**Figure 2:**
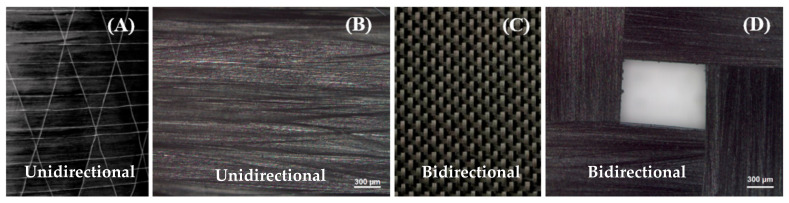
Images of (**A**,**C**) unidirectional and bidirectional carbon fabric and (**B**,**D**) their microscope images.

Portland cement (PC), grade CEM II/A-LL 42.5 N, and the quartz sand used in the present study, with particle sizes in the range of 0.1–0.5 mm, were purchased from a local company in Lithuania, JSC Akmenes Cementas. Both types of carbon fabrics with unidirectional and bidirectional structure were purchased from R&G Faserverbundwerkstoffe GmbH (Waldenbuch, Germany) and Otto Bock Healthcare GmbH (Duderstadt, Germany). The morphology and geometrical structure of both types are shown in Figure 2. They were composed of fabric with high fibre density distributed in a longitudinal and transverse direction, uniformly in the case of bidirectional fabric, with very small voids between the cross-linking fibre. The yarns were distributed in only one direction, with larger gap areas compared to bidirectional fabric; this the matrix to penetrate into these free spaces in the fabrics and achieve high adhesion between the cement fraction in the upper and lower components [3].

### 2.2. Preparation of CF/CM Samples

**Figure 3:**
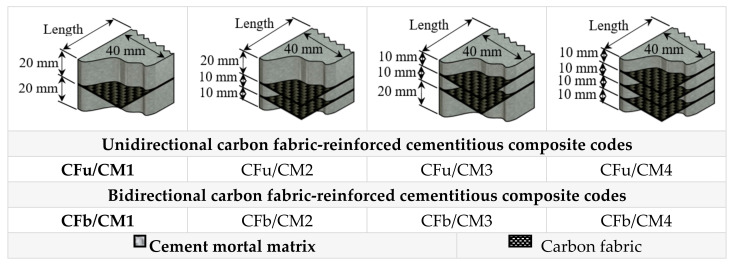
Configuration of carbon fabrics in the prepared matrices and their codes.

The preparation process was started with preparation of neat cementitious matrix (cement mortar: CM) sample with size of 40 mm × 40 mm × 160 mm for reasons of comparison [30,33]. The neat sample was prepared through mixing 1.25 wt.% of the super-plasticiser colloidal silica (refer to PC) with PC, followed by blending quartz sand with colloidal silica/PC at a ratio of 1.8 (sand/PC). After that, water was added to the mixture at a precise ratio (refer to PC fraction). Subsequently, the sample was cast in metal moulding, covered by plastic film, and left for 24 h for drying purposes [34]. Thus, after it was demoulded from the module, a fine aggregate cementitious composite (five samples from each batch) was prepared. In order to investigate the effect of embedding fabrics and their different structures on the flexural behaviour of CM, two different structures of fabric were involved. The matrices with different orientations (in particular, single, double, and triple layers) and each sample were assigned a code based on the fabric configuration and their structure in the matrix, as shown in Figure 3.

In a similar manner, CF/CM samples were prepared. This was only carried out after including CF and stretching it slightly on a 10 or 20 mm thick layer of fresh CM (bottom, upper, and middle) and levelling it with a metal trowel. This was followed by a drying process, as before. Then, the sample was left in a moist chamber at 20 °C and 100% relative humidity for curing purposes. After 28 days, the specimens were removed from the chamber to be ready for future testing processes. It is worth mentioning that five specimens were prepared from each batch to check reproducibility of the results. Finally, the flexural results in the following sections were expressed as the average values of these results.

### 2.3. Characterizations of CF/CM Samples

An optical microscope was used to observe the morphology of the damage surfaces (cross-section and in-plan) and coherence of CF and cementitious matrix. The bending tests for CF/CM samples were performed using universal testing machine (Zwick Roell model) according to standard EN 196-1 (European Standard EN 196) and loading speed of 0.5 mm/min at room temperature [35,36]. The flexural stress ($\sigma_{f}$), strain ($\varepsilon_{f}$) and modulus ($E_{f}$) were calculated using Equations (1)–(3) based on the measured flexural data and sample geometry, in particular, flexural load (F), span length (L), width (b), thickness (t), and deflection (δ) [37]. Also, the universal testing machine was used to measure the compressive strength of the CF/CM samples, which is required as an input parameter in the modelling section using FEM, as shown later.

(1) $$\sigma_{f}=\frac{3FL}{2bt^{2}}$$

(2) $$\varepsilon_{f}=\frac{6\delta t}{L^{2}}$$

(3) $$E_{f}=\frac{FL^{3}}{4\delta bt^{2}}$$

### 2.4. Modelling of Ductility of CF/CM Samples

**Figure 4:**
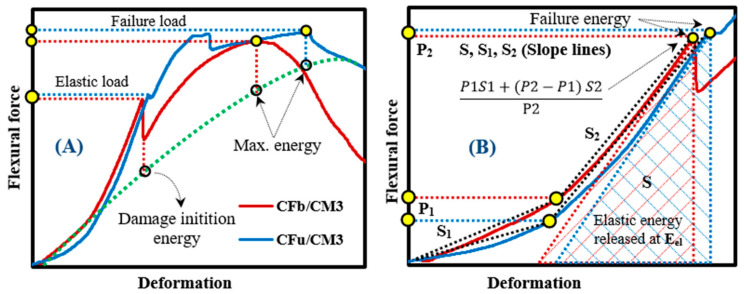
Schematic drawing of (**A**) flexural load–deflection curve’s characteristics and (**B**) estimation of all energies, including elasticity energy of CF/CM sample.

The ductility structure of CF/CM samples up to the end of the fracture was modelled based on energy criteria using the models of Naaman (1995) and Grace et al. (1998) [38,39]. In this kind of modelling, ductility is expressed as a dimensionless parameter, named ductility index (DI), which can be calculated based on curvatures and rotations or reflections of beams, with very accurate results. Also, these models do not need to yield, and the ultimate strains of the material do not occur in brittle structures like in the present research. In this regard, the DI of CF/CM samples was defined based on the total energy (Etotal), elastic energy (Eelastic), and failure energy (Einelastic) using Equations (4) and (5) [38,39]. All of these kinds of energies can be calculated from the measured flexural load–deflection curves, where Etotal represents the area under the curve up to the full break point (point). Also, Etotal is a sum of elastic energy (area of the triangle formed at the fracture load) and failure energy (difference between the total and elastic energy). In addition, elastic energy can be estimated as the slope of two initial straight lines of the measured flexural load–deflection curves, as shown in Figure 4 [30], which was presented based on the measured flexural data (Section 3.3).

(4) $$DI\;\left(\mathrm{Naaman}\right)=\frac{1}{2}\left(\frac{E_{total}}{E_{elastic}}+1\right)$$

(5) $$DI\;\left(\mathrm{Grace’s}\right)=\frac{E_{in-elastic}}{E_{total}}$$

## 3. Results and Discussions

### 3.1. Characteristics and Features of the Fracture CF/CM Panel Surfaces

**Figure 5:**
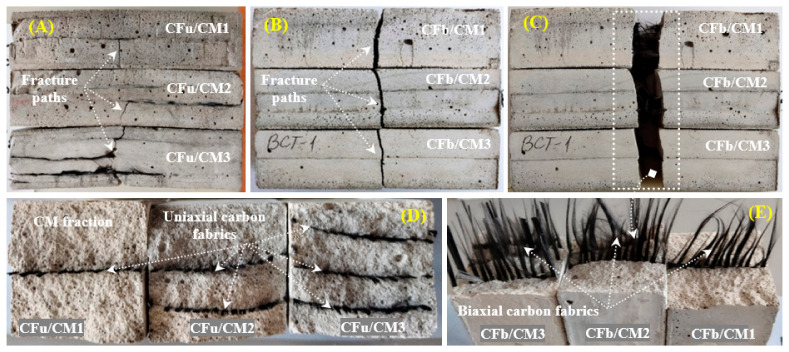
Photographs of (**A**–**C**) fracture paths of CF/CM specimens and (**D**,**E**) cross-section features of CF/CM specimens.

Figure 5 shows the surface and cross-section photographs of the fractured CFu/CM and CFb/CM specimens by the end of the flexural test. As shown, all the tested specimens were completely collapsed under the applied flexural load, and the failure started from the bottom surfaces. However, the fracture paths and their mechanism were changed depending on the geometry of the used fabrics and their amount in the matrices. In the case of the CFu/CM specimens, the fracture paths (from the surface front view) occurred at the middle in the form of several vertical fractures (parallel to the direction of applied load) and some horizontal fracture (in the form of detached surfaces against unidirectional carbon fabrics) (Figure 5A). Also, the failure was accompanied by too much tortuosity, especially at a higher amount of fabric, leading to the failure occurring in several steps in different inclined planes and fracture paths of the matrix segments. In addition, a slightly higher resistance to break and coherence between both fractions was observed. In the case of the CFb/CM samples, however, the fracture happened in the middle in all specimens and demonstrated a fracture shape that was almost vertical, even with changes in the number of fabrics that were included in the composite. This is a typical fracture shape in brittle cement materials (Figure 5B,C), whereas the cross-section views of the CF/CM panels show (Figure 5D,E) a uniform distribution and geometry of both types of carbon fabrics in the fabricated composites with strong coherence. Some corrugation was also observed in the fabrics used on damaged surfaces; this was produced during the fabrication and the hardening processes of this tight-casting mould.

### 3.2. Morphology of the Damaged Surfaces

**Figure 6:**
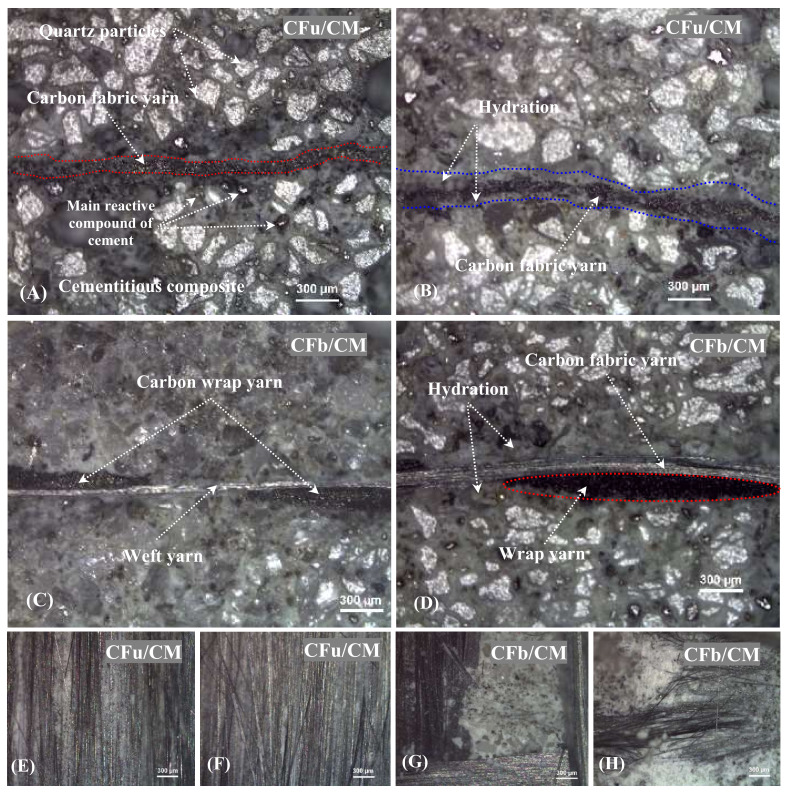
Cross-sectional morphology images of (**A**–**D**) CF/CM samples and (**E**–**H**) surface morphology of the detached CF/CM specimens.

The damaged cross-section surfaces of CF/CM composites specimens were examined using an optical microscope to observe changes in their morphology and the carbon fabrics that were incorporated into the cementitious composites. Figure 5 shows the microstructure images of damaged cross-section surfaces after cutting them using a diamond tool, polishing them, and coating them with a thin gold film. The observation process was performed on two samples: one included a unidirectional carbon fabric, and the other included a bidirectional carbon fabric to avoid repetitions. The observation process indicated that quartz particles (white dots) were distributed regularly, and both types of carbon fabrics were fully incorporated into the fabricated composites. Thus, the composites were fabricated under ideal conditions. Also, it was observed that in the case of unidirectional fabrics (Figure 6A,B), the damage samples contained only small dots distributed uniformly in the horizontal plan and referred to unidirectional longitudinal yarn’s (wrap yarn) cross-section geometry (a similar structure to that observed in the raw material carbon fabric). The similar structure was obtained in the case of bidirectional carbon fabrics (Figure 6C,D) but with an additional, very thin transverse yarn (weft yarn); this is a property of this kind of fabric, as listed earlier. In addition, the outer surface of both types of threads is covered with a thick layer of soluble cement molecules containing belite (C2S) and alite (C3S) phases [40] in addition to calcium silicate hydrate gel (CSH), ferrite (C4AF), and calcium hydroxide (CH) phases (the blue lines and light-grey areas in the figure below), which are considered the main elements in cement hydration products and which control the behaviour of the fabricated matrices [17]. Also, the surface morphology of the fabric inside the selected matrices was observed after mechanically separating the fabrics from the cementitious composite. It was observed that after the separation process, some fabrics were still left on the separate surfaces or were completely removed; in some cases, the fabrics were damaged. Therefore, the examination process, using a microscope, was performed on the detached upper and lower surfaces in order to better understand and check the incorporation of fabric and its bonding in the horizontal planes (in-plan).

Figure 6E–H display the surface morphology images of the detached segment from the CFu/CM and CFb/CM specimens. As shown, in the case of unidirectional samples, the longitudinal thread (the wrap yarn was “black needle-like”) remained stuck on the separated upper and lower surfaces almost equally. This was due to the high surface contact between the yarn and cementitious hydration products [41]; this increased their cohesion and strength as well. On the other hand, in the case of bidirectional carbon fabrics, three main features were observed. Yarn was distributed in the longitudinal and transverse directions, with almost the same density in both directions, with overlapping or nodes (joined together using friction bond). Between these yarns, several grey gaps appeared; these were related to the cementitious part. Generally, hydration products can cover and be in contact with the outer surfaces of longitudinal and transverse yarns. But these products did not have the opportunity to penetrate the nodes and create a strong bonding with their internal surfaces [42], thereby obstructing the hydration process and exerting an effect on its mechanical performance, as shown in the next results. Also, it was noted that the yarns were completely damaged; in other words, both the cementitious matrix and the fabric were damaged.

### 3.3. Flexural Strength of CF/CM

**Figure 7:**
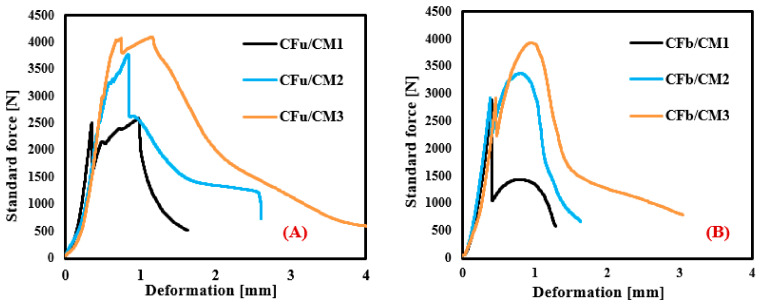
Flexural load–deformation profiles of (**A**) CFu/CM, and (**B**) CFb/CM specimens.

Figure 7A,B and Table 1 show the effect of introducing carbon fabric (and its geometry and amount) on flexural load–deflection profiles and mechanical measurements of the CFu/CM and CFb/CM specimens. Compared with the CM sample (which has a linear relationship with the combined elastic and plastic zones), the shape of the mechanical profiles of both CF/CM samples changed from that of a linear relationship to one where there was no longer any failure in the two different regions with different features. The first region refers to the elastic zone (uncracked panel), which is characterised by its linear relationship in both samples. However, it was observed that the final flexural load values of this region were increased significantly by increasing the number of unidirectional fabrics used in the matrix from 2456 N (CFu/CM1) to 4034 N (CFu/CM3): a 64% improvement was produced (Figure 7A). Subsequently, a gradual reduction in the load occurred (cracking that extended to the end of the plastic region and fracture) until the next fabric layer and then increased again, with almost the same maximum strength values as the elastic zone. Similarly, in the case of the CFb/CM specimens (Figure 7B), two peak points were obtained (until the end of the elastic zone and at the break). The effect of fabric strengthening was more pronounced in the case of the unidirectional fabric combined with activation in the tension component; this sample demonstrated a great deal of resistance to bending. However, it was observed that the number of fabrics introduced into the matrix did not have an effect on the load value (2853 N) at the end of the elastic zone, and the main influence was manifested in the maximum value in the plastic zone, which was estimated at 2881 N (CFb/CM1), 3370 N (CFb/CM2), and 3924 N (CFb/CM3). All these features confirm that the failure changed from a brittle to a ductile type in both samples. As shown, both samples have flexural load values that are almost similar, with deformations at break that are almost similar but that are still higher than those of the neat sample due to the high strength and flexibility of the carbon fabrics used. In addition, the transverse threads in the CFb/CM samples did not have a major effect on the load compared with the effect of the longitudinal threads because these threads were parallel to the applied flexural testing load [41]. In addition, these samples improved the C-S-H bonds and hydration performance of the fabricated composite [17]. However, it was observed that the mechanical profile of the CFb/CM specimens is very smooth compared to that of the CFu/CM specimens due to the regular geometry of yarns in the longitudinal and transverse directions, which produces an equality in loading conditions in both directions, resulting in sharp curves. Furthermore, the empty spaces in bidirectional carbon fabrics allows the casted mortar to flow through them; this helps the fabrics to cohere strongly with mortar particles, leading uniform bonding and smoothing results. Meanwhile, the overlapping between longitudinal and transverse threads impedes hydration products at these nodes; this effects the maximum load in general. On the other hand, unidirectional fabric has threads in only one direction, with different loadings in both directions. Finally, some deformations developed in the main failure region due to the nature of the plasticity property of the specimens and the effect of the residual loading, especially in the case of the unidirectional fabric, due to its higher flexibility [43,44]. Compared with the results for glass fibre-reinforced mortar (2788 N) [29], the carbon fibre-reinforced mortar showed higher flexural load (4034 N), with a 45% improvement.

### 3.4. Calculations of Flexural Energies

**Figure 8:**
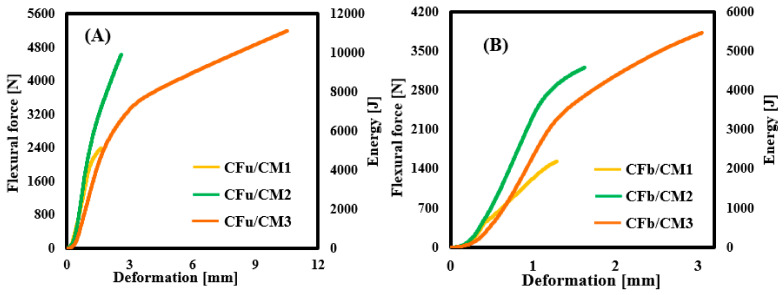
Total flexural energy-deformation profiles of (**A**) CFu/CM and (**B**) GFb/CM specimens.

The flexural energies (*E_total_*, *E_elastic_*) required in order to calculate the DI term using the Naaman and Grace models were determined in this section; these energies were based on the flexural energy-deformation curves of each batch. The integration of the area under the curves represents the value of *E_total_*, and their equivalent values until reaching break points are shown in Figure 8A,B. As shown in the CFu/CM curves, the maximum flexural energy was estimated at 2400 J (CFu/CM1), 4640 J (CFu/CM2), and 11,123 J (CFu/CM3). In the case of CFb/CM specimens, *E_total_* was estimated at 1531 J (CFb/CM1), 3212 J (CFb/CM2), and 5467 J (CFb/CM3). This means that the *E_total_* was increased significantly by embedding the carbon fabric (it was increased by 363% (CFu/CM) and 257% (CFb/CM)) due to the high adsorption energy of the carbon fibre and its strong role in delaying crack propagation [45,46]. Meanwhile, the *E_elastic_* term was determined, as noted before, depending on the average slope of the first two linear relationships in the flexural load–deflection profiles. Follow that, we calculated the $E_{in-elastic}$ term as the difference between the *E_total_* and *E_elastic_* of each specimen.

### 3.5. Evaluation of Ductility Index

**Figure 9:**
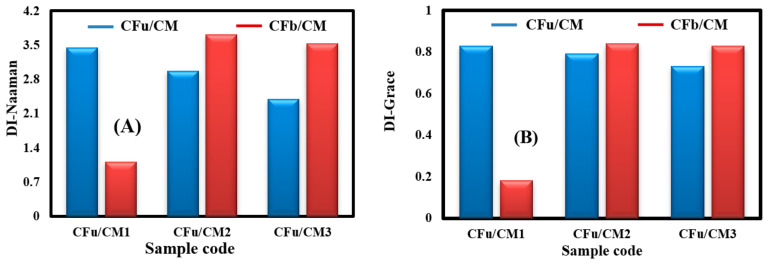
The DI-Naaman and DI-Grace of (**A**) CFu/CM and (**B**) CFb/CM specimens.

Based on the calculated flexural energies in the above section, DI-Naaman and DI-Grace were determined using Equations (4) and (5), and the results are shown in Figure 9. The results show that the DI of the CFu/CM specimens was decreased by adding more carbon fabrics, while the DI of the CFu/CM specimens remained higher than that of the neat mortar sample, with an improvement that reached as high as 140%. On the contrary, in the case of the CFb/CM specimens, the DI increased by embedding fabrics (and increased significantly in the case of the CFb/CM2 sample) and then decreased slightly in the case of the CFb/CM3 sample as a result of a decrease in the inelastic energy term. These results confirm that the mode of behaviour of both matrices was changed from brittle behaviour into a ductile mode after introducing the fabrics, especially in the case of the bidirectional carbon fabrics. This means that both batches had a high ability to absorb inelastic energy while retaining the same loading capacity, where ductility is directly proportional to the inelastic segment. However, the CFb/CM2 sample showed a high ductility performance: the prepared matrix absorbed more elastic energy, reducing carbon fibre splitting along the longitudinal and transverse directions, which would otherwise fully damage the cement matrix segments and be followed by carbon fibre rupture. This also showed that the DI of metrics can be controlled by the location of the fabrics in the matrix and by the fabrics’ geometry.

### 3.6. Energy-Based Model to Predict the Absorbed Energy During the Flexural Test

**Figure 10:**
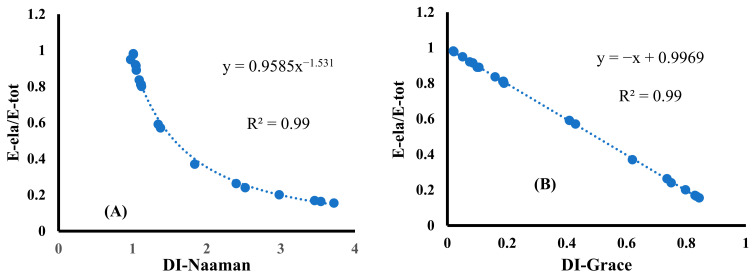
A normalised energy fitting for CF/CM specimens using (**A**) Naaman and (**B**) Grace models.

In order to formulate an empirical equation for predicting the ductility of CF/CM, a normalised reinforcement ratio–DI relationship was plotted [47]. Figure 10 displays plots for normalised energy compared to the DI-Naaman and DI-Grace values for all CF/CM specimens used in the present research. It was noted that the power regression and linear relationship were an ideal curve fitting for the Naaman and Grace models, respectively. Based on that, the empirical formula for predicting $E_{in-elastic}$ absorption was formulated as illustrated in Equation (6). This formula can be used to calculate an approximate value of $E_{in-elastic}$ absorption of CF/CM specimens, while Eela and DI can be obtained from flexural experiments.

(6) $$E_{in-ela}=E_{ela}\left(DI^{1.531}-1\right)$$

## 4. Conclusions

In this work, the flexural performance of carbon fibre-reinforced mortar with different geometries (unidirectional and biaxial) and distribution (one to three layers) was studied. Also, the effect of fabric geometries and orientation on the ductility index of CF/CM matrices was determined using different two methods of energy consumption principles. The flexural results showed that the unidirectional and bidirectional matrices followed a roughly similar trend, where the flexural load increased gradually (and approximately at the same rate (3924–4034 N)) as the number of fabrics involved increased. However, the bidirectional matrices showed higher deformation, with very smooth failure curves with only one maximum peak, unlike unidirectional matrices, which showed two peaks. On the other hand, bidirectional matrices showed higher deformation, with very smooth failure curves with only one maximum peak, in contrast to unidirectional matrices, which showed two peaks, especially in cases where three fabrics were used. The bidirectional matrices also showed a higher ductility index (140% improvement) compared to the pure mortar sample. Accordingly, the developed carbon fabric-reinforced mortar composites (3 layers) can be used in applications requiring high strength and deformation with high predictability of failure. CF/CM matrices can enhance the sustainability of living walls by improving the safety of the infrastructure through improving its ductility. Also, the developed CF/CM composites can be used in critical applications subjected to dynamic loads, explosions, and earthquakes. Finally, carbon fabric is expensive, which may affect the economic performance of the developed CF/CM composites; thus, other sources, such as recycled fabric from wind turbines, must be sought to obtain cheaper carbon fabric [48].

## Figures and Tables

**Table 1:** Flexural characteristics of CFu/CM and CFb/CM specimens.

Mechanical Parameters	CM	Unidirectional Glass Fabric			Bidirectional Glass Fabric		
		CFu/CM1	CFu/CM2	CFu/CM3	CFb/CM1	CFb/CM2	CFb/CM2
Load at break (N)	2337	2583	3764	4092	2881	3370	3924
Stress at break (MPa)	5.6	6.05	8.82	9.59	6.75	7.90	9.20
Deformation at break (mm)	0.21	0.98	0.84	1.16	0.4	0.83	0.98
Young’s modulus (GPa)	0.78	0.68	0.69	0.67	0.67	0.79	0.57

## Data Availability

The original contributions presented in this study are included in the article. Further inquiries can be directed to the corresponding author.
